# Changes in State-Level Cigarette Sales During the COVID-19 Pandemic

**DOI:** 10.1001/jamanetworkopen.2022.48678

**Published:** 2022-12-28

**Authors:** Samuel Asare, Zheng Xue, Anuja Majmundar, Priti Bandi, Nigar Nargis

**Affiliations:** 1 Tobacco Control Research, Surveillance & Health Equity Science, American Cancer Society, Kennesaw, Georgia; 2 Risk Factors & Screening Surveillance Research, Surveillance & Health Equity Science, American Cancer Society, Kennesaw, Georgia

## Abstract

This cohort study estimates state-level changes in cigarette sales in the US during the COVID-19 pandemic.

## Introduction

Cigarette sales in the US were higher than expected after the onset of the COVID-19 pandemic in March 2020,^1,2^ but state-specific changes are unknown. This study estimated changes in state-level cigarette sales during the pandemic.

## Methods

We used state-level aggregate cigarette sales volumes released for consumption by tobacco manufacturers and importers based on their monthly filings to the US Department of Treasury from quarter 3 of 2008 (2008Q3) to 2021Q4. The outcome was state-level quarterly cigarette sales volumes converted into packs (20 sticks) per capita sales based on state annual population data.^3^

Interrupted time series analysis was used to estimate changes in cigarette sales before (2008Q3-2020Q1) and after (2020Q2-2021Q4) the onset of the pandemic after accounting for inflation-adjusted cigarette prices, quarterly seasonality, and self-reported sociodemographic characteristics^4^ (eMethods in Supplement 1). Expected cigarette sales after the onset of the pandemic were extrapolated from the state-level prepandemic trend. Changes in cigarette sales after the onset of the pandemic were calculated as the mean difference between observed and expected quarterly cigarette sales. National-level estimates were calculated based on estimates from all states and Washington, DC. This cohort study followed the STROBE reporting guideline. The study used state-level aggregate cigarette sales data and did not require institutional review board approval or informed consent according to the US Department of Health and Human Services (45 CFR §46).

Analyses were conducted using Stata, version 17.0 (StataCorp LLC), and all *t* tests were 2-sided, with *P* < .05 considered statistically significant.

## Results

The sample for analysis consisted of 2754 observations over 54 quarters. National-level cigarette sales per capita were higher in the prepandemic vs pandemic period, while the inflation-adjusted cigarette price increased between the 2 periods. The percentage of persons aged 65 years or older, with college degrees, male, with household incomes lower than $10 000 and $150 000 or more, unmarried, and who were Asian was higher; those younger than 65 years, without college degrees, female, with household income from $10 000 to $149 999, and married was lower during the pandemic (Table).

**Table.** Differences in Cigarette Sales and Covariates Before and After the Onset of the COVID-19 Pandemic^a^

Variable	%, Mean (SD)			*P* value
	Before COVID-19 pandemic (2008Q3–2020Q1)	After the onset of COVID-19 pandemic (2020Q2–2021Q4)	Difference, After – Before	
Observations, No.	2397	357	2754	NA
Quarterly packs of cigarette sales, packs	11.81 (5.22)	9.18 (4.35)	−2.62 (0.29)	<.001
Price per pack of cigarettes, Feb 2020, $	6.58 (1.58)	7.51 (1.63)	0.93 (0.09)	<.001
Age, y				
<25	31.48 (2.99)	28.78 (3.15)	−2.70 (0.17)	<.001
25-44	25.38 (2.46)	24.90 (2.71)	−0.48 (0.14)	<.001
45-64	27.26 (2.33)	25.96 (2.01)	−1.30 (0.13)	<.001
≥65	15.88 (2.78)	20.36 (3.06)	4.48 (0.16)	<.001
Educational level				
Less than high school	14.97 (3.18)	11.99 (2.36)	−2.98 (0.18)	<.001
High school graduate	29.30 (4.29)	28.37 (4.67)	−0.93 (0.25)	<.001
Some college	27.35 (3.89)	26.44 (4.14)	−0.91 (0.22)	<.001
College or higher	28.39 (6.77)	33.21 (7.51)	4.82 (0.39)	<.001
Gender				
Male	48.64 (1.13)	48.82 (1.12)	0.19 (0.06)	.003
Female	51.36 (1.13)	51.18 (1.12)	−0.19 (0.06)	.003
Household income, $				
<10 000	20.34 (5.53)	22.08 (4.87)	1.73 (0.31)	<.001
10 000-29 999	16.84 (4.39)	11.78 (3.12)	−5.06 (0.24)	<.001
30 000-59 999	23.66 (3.35)	20.44 (3.54)	−3.23 (0.19)	<.001
60 000-149 999	30.89 (5.13)	33.04 (4.40)	−2.15 (0.29)	<.001
≥150 000	8.27 (4.42)	12.67 (5.17)	4.40 (0.26)	<.001
Marital status				
Married	52.69 (4.06)	52.17 (3.57)	−0.52 (0.23)	.02
Unmarried	47.31 (4.06)	47.83 (3.57)	0.52 (0.23)	.02
Race and ethnicity^b^				
Asian	4.05 (5.91)	4.76 (6.32)	0.71 (0.34)	.04
Black	10.31 (10.14)	9.97 (9.36)	−0.34 (0.57)	.55
White	81.21 (13.07)	80.83 (12.58)	−0.38 (0.57)	.61
Other	4.43 (5.62)	4.44 (4.97)	0.01 (0.31)	.98

Abbreviation: NA, not applicable.

^a^ Cigarette sales and prices were obtained from the US Department of Treasury based on July 2008 to December 2021 monthly filings by tobacco manufacturers and importers. Data on the composition of age, educational level, gender, household income, marital status, and race and ethnicity were sourced from the Basic Monthly Current Population Survey of the Census Bureau. Individual-level sociodemographic characteristics in each state and month were collapsed into the state-by-quarter proportion of individuals in each category (compositions) and then linked to the state-level quarterly cigarette sales data.

^b^ Race and ethnicity were controlled for in the models to account for differential cigarette consumption patterns across races and ethnicity whose composition changed over time within states. Other races and ethnicities, as categorized in the US Census Bureau Basic Monthly Current Population Survey, included American Indian, Alaska Native only, Hawaiian/Pacific Islander only, and multiple races.

Compared with expected sales, quarterly cigarette sales per capita were lower in 8 states and Washington, DC, higher in 22 states, and unchanged in 20 states during the pandemic. Colorado experienced the highest absolute decrease (−0.88 packs; 95% CI, −1.1 to −0.7 packs); New Hampshire had the highest absolute increase (2.3 packs; 95% CI, 1.2-3.4 packs) in quarterly per capita packs of cigarette sales. Washington, DC, had the highest percentage decrease (18.6%; 95% CI, −34.4% to −2.8%); Pennsylvania had the highest percentage increase (16.7%; 95% CI, 7.6%-25.9%) in quarterly packs of cigarette sales. Quarterly national-level cigarette sales increased by 0.23 packs (95% CI, 0.15-0.31 packs) per capita, corresponding to a 1.9% (95% CI, 1.0%-2.9%) increase in sales (Figure).

**Figure.**
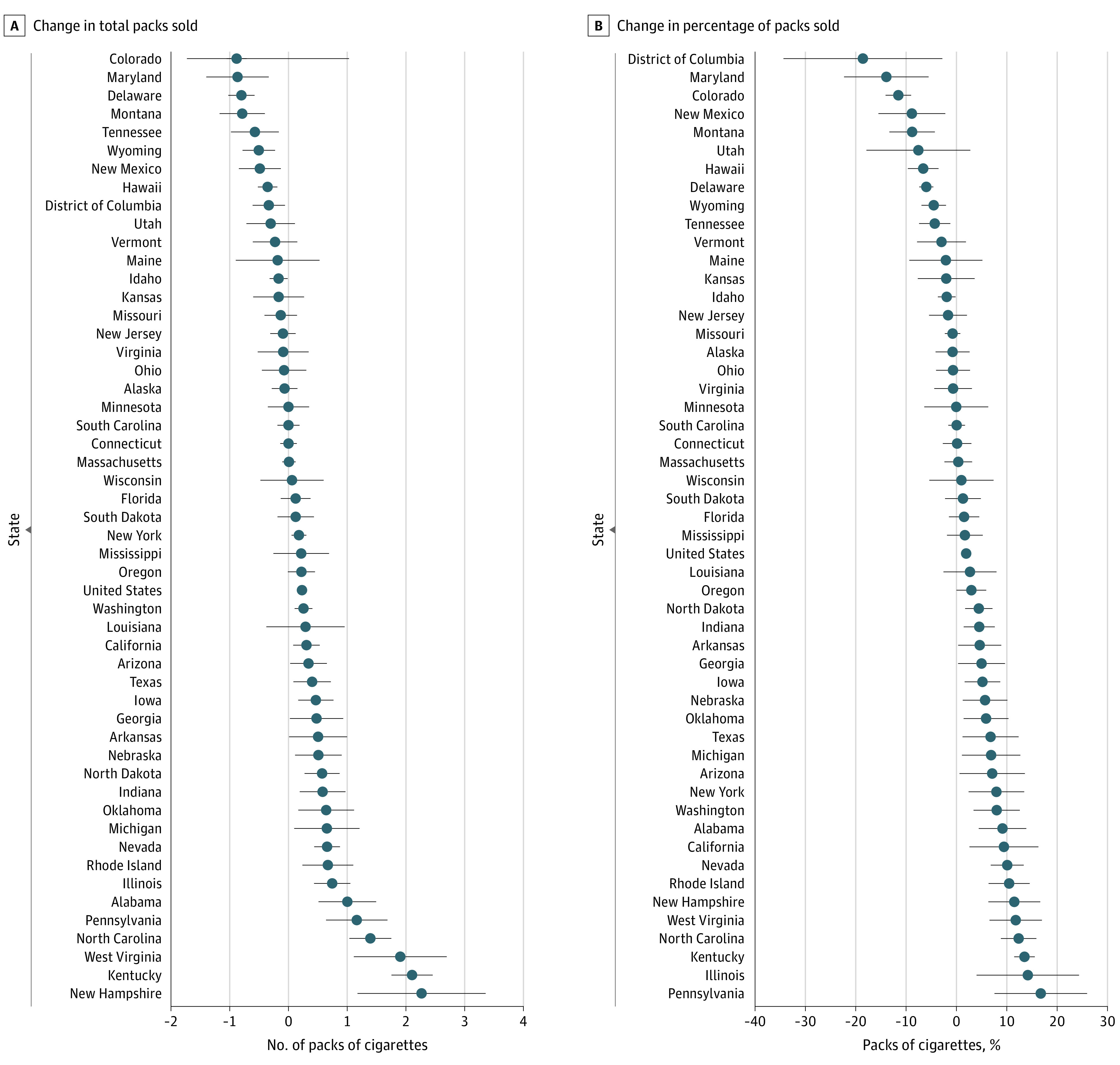
Mean Difference Between Observed and Expected Quarterly Cigarette Sales per Capita The mean difference between the observed and expected quarterly cigarette sales per capita is expressed as a count of packs (A) and a percentage change from the expected sales (B). The point estimates (circles) with 95% CIs (error bars) are the mean difference between the observed and expected quarterly cigarette sales per capita based on the pre–COVID-19 trends. The expected cigarette sales after the onset of the COVID-19 pandemic were estimated based on an interrupted time series model described in the eMethods in Supplement 1.

## Discussion

The national estimate of a 1.9% increase in sales is consistent with earlier findings of increases in cigarette and cigar sales in the US during the pandemic.^1,5^ The differential changes in cigarette sales across states may reflect state-level variation in the COVID-19 pandemic response and tobacco control policy environment. Limitations of the study are that actual cigarette consumption could differ from cigarette sales, no control group was used since all states were affected by the pandemic, and March 2020, marking the beginning of the COVID-19 pandemic, was included in the prepandemic period. States with increases in cigarette sales over the expected sales could experience a slowdown in the previous downward changes in cigarette consumption, which may reverse the progress in tobacco control and need to tighten tobacco control policies to achieve prepandemic targets.

## Notes

Supplement 1. **eMethods.** Interrupted Time Series Analysis
eReferences

Supplement 2.
Data Sharing Statement
