# Supplementary material for: Changes in State-Level Cigarette Sales During the COVID-19 Pandemic
Source: JAMA Netw Open. 2022 Dec 28;5(12):e2248678. doi: 10.1001/jamanetworkopen.2022.48678 (PMC9857347; doi:10.1001/jamanetworkopen.2022.48678)
Supplement: Supplement 1. — eMethods. Interrupted Time Series Analysis eReferences [file jamanetwopen-e2248678-s001.pdf]

Supplementary Online Content

Asare S, Xue Z, Majmundar A, Bandi P, Nargis N. Changes in state-level cigarette sales during the COVID-19 pandemic. *JAMA Netw Open*. 2022;5(12):e2248678. doi:10.1001/jamanetworkopen.2022.48678

eMethods. Interrupted Time Series Analysis

eReferences

This supplementary material has been provided by the authors to give readers additional information about their work.

© 2022 Asare S et al. *JAMA Network Open.*

eMethods

Interrupted Time Series Analysis

The expected cigarette sales were calculated based on the interrupted time series equation:

$$Y_t = \beta_0 + \beta_1 T_t + \beta_2 X_t + \beta_3 X_t \times T_t + \varphi \mathbf{Z}_t + \gamma_{\mathbf{m}} + \xi_t,$$

where Y_t = per capita quarterly cigarette sales in packs, X_t = an indicator variable taking the value 0 before the onset of the COVID-19 pandemic from quarter 3 of 2008 (2008Q3) to quarter 1 of 2020 (2020Q1) and 1 starting from quarter 2 of 2020 (2020Q2) to the last quarter of 2021 (2021Q4), T_t = linear time trend in cigarette sales, which takes 0 starting from 2008Q3 and increases by 1 every quarter interval, $\boldsymbol{Z}_t$ = a vector of covariates adjusted for in the model (inflation-adjusted cigarette price and composition of age, education, gender, household income, marital status, and race and ethnicity), and γ_m = a vector of quarter fixed effects, which included 3 dummy variables, one for each quarter from Q2 to Q4 and omitted Q1 as the reference period. The coefficients β_1 = slope of cigarette sales before the onset of the COVID-19 pandemic, β_2 = change in the level of cigarette sales immediately after the onset of the pandemic, and β_3 = change in the slope of cigarette sales after the onset of the pandemic. In states where the trends in cigarette sales before the onset of the COVID-19 pandemic were not constant, we also accounted for any changes in slope or intercept. The expected sales from 2020Q2 to 2021Q4 were extrapolated based on the pre-pandemic trends (2008Q3 to 2020Q1). Changes in cigarette sales after the onset of the COVID-19 pandemic were calculated as the mean difference between the observed and expected quarterly cigarette sales. The national-level estimate was calculated based on the estimates from all states and Washington, DC.

© 2022 Asare S et al. *JAMA Network Open.*

Data was available for all states and Washington, DC. We estimated the model for each state separately using the “regress” command in Stata 17.0 (StataCorp LLC). For Massachusetts and its bordering states, we first estimated changes in cigarette sales associated with the Massachusetts comprehensive menthol flavor ban, implemented in June 2020, before estimating changes in cigarettes sales after the onset of the COVID-19 pandemic. Previous studies have shown that the Massachusetts menthol flavor ban was associated with reduced cigarette sales in the states[1] and a slight increase in sales in its bordering states.[2]

We obtained the information on prices per pack (20 sticks) of cigarettes for each state and month from the US Department of Treasury. We converted the state-level cigarette prices in each month into February 2020 constant prices using consumer price indices from the US Bureau of Labor Statistics.[3] The state-level inflation-adjusted cigarette prices available in each month were averaged into state-level prices per pack of cigarettes in each quarter. We then included the continuous variable in the models.

Data on the population percentages by age, education, gender, household income, marital status, and race and ethnicity were estimated from the individual-level self-reported data collected in the Basic Monthly Current Population Survey of the Census Bureau.[4] We collapsed the individual-level socio-demographic characteristics in each state and month into the state-by-quarter proportion of individuals in each category (compositions) and linked them to the state-level quarterly cigarette sales data.

© 2022 Asare S et al. *JAMA Network Open.*

eReferences

1. Asare S, Majmundar A, Westmaas JL, et al. Association of Cigarette Sales With Comprehensive Menthol Flavor Ban in Massachusetts. *JAMA Internal Medicine*. Published online January 4, 2022. doi:10.1001/jamainternmed.2021.7333

2. Asare S, Majmundar A, Westmaas JL, et al. Spatial Analysis of Changes in Cigarette Sales in Massachusetts and Bordering States Following the Massachusetts Menthol Flavor Ban. *JAMA Network Open*. 2022;5(9):e2232103. doi:10.1001/jamanetworkopen.2022.32103

3. Consumer Price Index Home : U.S. Bureau of Labor Statistics. Accessed November 4, 2022. https://www.bls.gov/cpi/

4. Census Bureau. Basic Monthly Current Population Survey. The United States Census Bureau. Accessed June 5, 2022. https://www.census.gov/data/datasets/time-series/demo/cps/cps-basic.html

© 2022 Asare S et al. *JAMA Network Open.*
