# Supplementary material for: Changes in State-Level Cigarette Sales During the COVID-19 Pandemic
Source: JAMA Netw Open. 2022 Dec 28;5(12):e2248678. doi: 10.1001/jamanetworkopen.2022.48678 (PMC9857347; doi:10.1001/jamanetworkopen.2022.48678)
Supplement: Supplement 2. — Data Sharing Statement [file jamanetwopen-e2248678-s002.pdf]

Data Sharing Statement

Asare. Changes in State-Level Cigarette Sales During the COVID-19 Pandemic. *JAMA Netw Open*. Published December 28, 2022. doi:10.1001/jamanetworkopen.2022.48678

Data
Data available: No

Additional Information
Explanation for why data not available: The data was provided directly by the US Department of Treasury and is not publicly available.
